# 3D Printed replica of articular fractures for surgical planning and patient consent: a two years multi-centric experience

**DOI:** 10.1186/s41205-016-0006-8

**Published:** 2016-12-01

**Authors:** Nicola Bizzotto, Ivan Tami, Attilio Santucci, Roberto Adani, Paolo Poggi, Denis Romani, Guilherme Carpeggiani, Filippo Ferraro, Sandro Festa, Bruno Magnan

**Affiliations:** 1 Orthopedic and Hand Surgery Department, Dolomiti Sportclinic, Via Purger 181, Ortisei-Bolzano, Italy; 2 Centro manoegomito, Clinica Ars Medica, Via Grumo 16, Gravesano - Lugano, Switzerland; 3 Orthopedic Department, Villa Stuart, FIFA Medical Center, Via Trionfale 5952, Roma, Italy; 4 Hand Surgery Department, University Hospital of Modena, Viale del Pozzo 71, Modena, Italy; 5 Radiology Deparment, Hospital of Lodi, Via Savoia 4, Lodi, Italy; 6 Orthopedic Deparment, University Hospital of Verona, Piazzale Stefani 1, Verona, Italy; 7 Tecs Italia Laboratory srl. Via A. Magio, 12, Bassano del Grappa, (Vi) Italy; 8 3DZ Industry: Via del Credito, 26/2, 3, Castelfranco Veneto, TV Italy

**Keywords:** Tibial Plateau, Radial Head, Distal Radius Fracture, Acrylonitrile Butadiene Styrene, Articular Fracture

## Abstract

**Background:**

CT scanning with 3D reconstructed images are currently used to study articular fractures in orthopedic and trauma surgery. A 3D-Printer creates solid objects, starting from a 3D Computer representation.

**Case Description:**

We report from two year of multicenter experience in 3D printing of articular fractures.

**Discussion and Evaluation:**

During the study period, 102 patients (distal radius fractures, radial head, tibial plateau, astragalus, calcaneus, ankle, humeral head and glenoid) underwent 3D printing. The medical models were used by surgeons to appreciate the dislocation of fragments and the yielding of the articular surface. In addition, models were showed to patient as part of the acquisition of the informed consent before surgery.

**Conclusions:**

3D printing of articular fractures are innovative procedures that achieve a preoperative tangible, highly useful evaluation of the fractures to plan intervention and educate patients.

## Background

3D printing is s a relatively low cost technology that uses a 3D computer representation (graphics or 3D virtual objects) to create solid replicas that can then be used for healthcare applications; 3D printing models of healthy or fractured bones are used in facial and neurosurgery to select locations of appropriate and optimal osteosynthesis, to study the appropriate fracture’s pattern and to reduce surgical time and improve outcomes in patients [3].

In orthopedic and trauma surgery actually, X-rays and Computed tomography (CT) with MPR (MultiPlanar Reformations) and 3D Volume Rendering are used to understand the dislocation of fragments, the amount of displacement and the joint involvement of articular fractures.

Very recently, with rapid distribution of commercial 3D printers within the hospital setting, orthopedic surgeons started to use 3D printed replica of pelvic fractures, acetabulum fractures [12], clavicle [6] and various articular fractures (like wrist [2], elbow, tibial plate…) to improve understanding of fracture by means of tactile and visual experience [1, 10]. Other pathologic conditions like spine disorders, dysplasia of hips or bone tumors are bone tumors are 3d-printed for surgical planning [9]. However, there is a paucity of publications that focus on a collection of patients. The purpose of this paper is to present our two-year multi-centric experience of 3D printed models of articular fractures in orthopedic and trauma surgery and hand surgery.

## Case Description

This study included six hospitals with subspecialized surgical services for trauma and/or hand procedures. The study period was January 2014 to December 2015, during which 102 patients (age range 20–78, 45 male and 57 female)) were enrolled. Written informed consent was obtained for each patient (for this research project).

The patients presented with the following fractures: distal radius (*n* = 31), tibial plateau (*n* = 19), radial head (*n* = 9), calcaneus(*n* = 15), astragalus (*n* = 5), ankle (*n* = 11), humeral head (*n* = 8) and glenoid (*n* = 4). A prerequisite for recruitment was that the patient is eligible for surgery (and hence has a clinical need for a medical model) because of fracture displacement, dislocation of the fragments, and/or instability.

All 102 patients underwent CT scan: Hitachi Presto (Hitachi Medical Corporation, Japan), Siemens Somatom (Siemens, Germany), GE Optima CT660 (GE Medical System, USA), Philips iCT256 (Philips, NL) situated in hospitals of our enrolled centers. Data was reconstructed at 0.625 mm increments with 0.625 mm reconstructions. Reconstructed DICOM images were uploaded into a OsiriX Dicom Viewer. Multiplanar Reformatting (MPR) and 3D Volume rendering of the fracture were obtained for diagnosis and assessing the anatomy for 3D printing. Working on the 3D-Volume Rendering Reconstruction, the fractured bone was isolated with digital scissor tool.

**Fig. 1:**
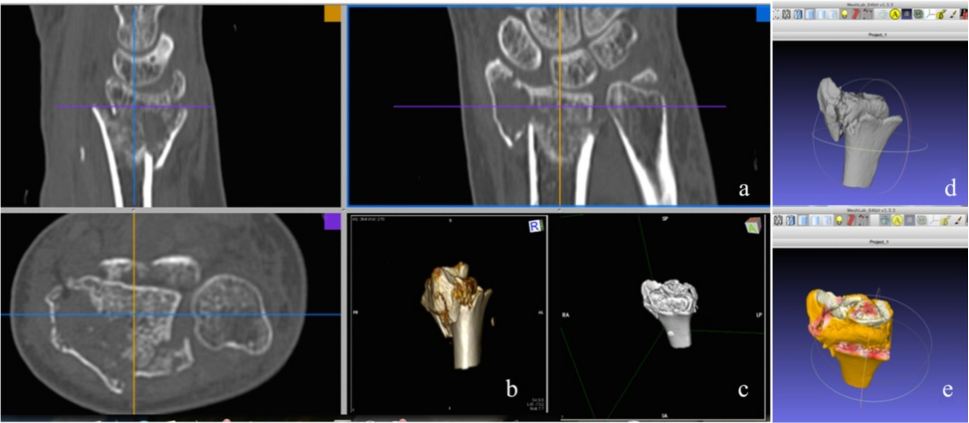
Creation of the .stl file and the color file of a distal radius fracture. **a** 2D Multiplanar Reconstructions; **b** 3D Volume Rendering; **c** Surface Rendering Mode, **d** Meshlab control; **e** model colored in Meshlab

Afterwards, with the “Surface Rendering” tool, the 3D model of fractured bone was created and exported to an .stl file. The file was analyzed and prepared for printing with Mesh Lab, a 3D dedicated software package. The models were manually colored: yellow was choose for the bone, red color lines were painted along the fractured fragments and white color was distributed over the joint surface area (where visible) (Fig. 1) under the supervision of a radiologist.

The models were exported in .obj and sent to a printer service near the hospitals. A post processing step (with a 3D Rendering Software) was sometimes necessary to create artificial bridges to connect serious displaced fragments to maintain the “anatomy” of the fracture. A ProJet 660 Color printer (3D Systems, Rock Hill, SC) was used to 3D print the models with gypsum-dust material. In our experience we support this material (instead of acrylonitrile butadiene styrene material, ABS) because it very realistically replicates bone.

## Results

The 1:1 models were printed in 4–8 h, depending of the anatomical regions. (4 h for a distal radius fracture and 8 h for a tibial plateau). The costs ranged from roughly $10USD for a model of the distal radius to $75USD for a tibial plateau.

**Fig. 2:**
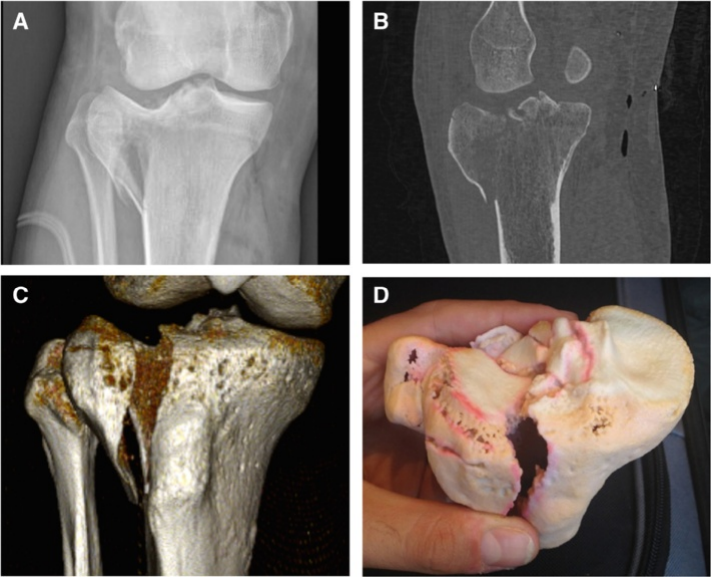
X ray (**a**), 2D CT Scan (**b**), 3D Volume rendering (**c**) and 3D printed replica (**d**) of a tibial plateau fracture of a 45 y old man after a motorbike injury

The printed fractured bone was handled prior by the surgeons, by residents and later by the patient, to be studied and examined (Fig. 2).

With the models in hands, the surgeons evaluated details as joint fragmentation, yielding and dislocation of the articular surface in a realistic way; these were presented to young residents and to medical-school-students to improve awareness of a trauma and fractures.

Generally we noticed that 3d printed models reproducing extra-articular/non-displaced fractures are not very advantageous compared to models of complexes/serious displaced fractures. Surgeons rated the use of models most beneficial for articular fractures with articular gaps or steps of 2 mm, or with a multi-fragmentary pattern (i.e., AO Classification type B and C for articular fractures); for simple and methaphyseal fractures (i.e., AO Classification type A) the models were not useful.

**Fig. 3:**
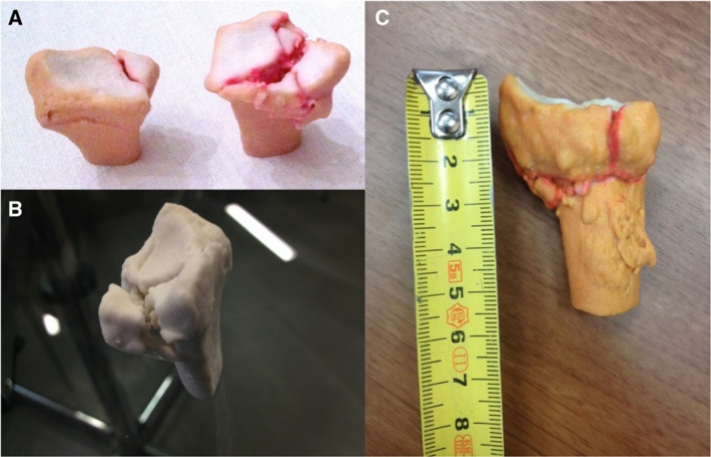
3D Printed Replica of Distal Radius fractures: examples. Color models (**a**), without colors (**b**), realistic measure (**c**)

**Fig. 4:**
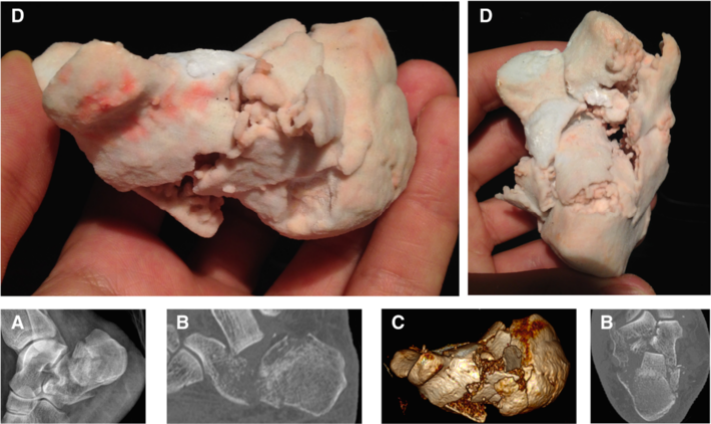
X ray (**a**), CT Scan (**b**), 3D Volume rendering (**c**) and 3D printed replica (**d**) of calcaneus fracture

**Fig. 5:**
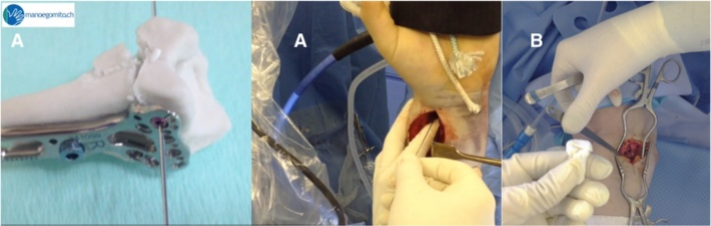
Plate and screw testing on a distal radius fracture (**a**). Radial head fracture with sterile model (**b**)

**Fig. 6:**
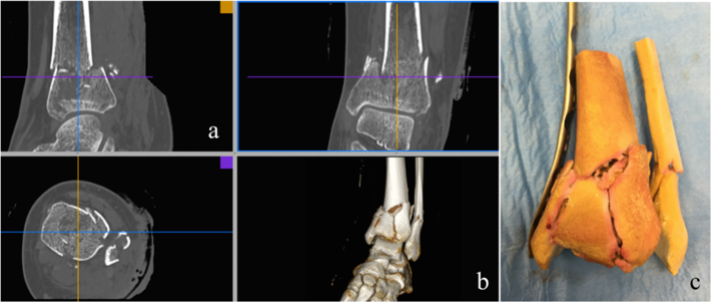
2D CT scan (**a**), 3D Volume rendering (**b**) 3D Printed Replica of an ankle fracture with sterile model on the surgical table for plate testing (**c**)

The day before going in operating theatre, the replica of distal radius fracture (Fig. 3), calcaneus (Fig. 4), ankle, radial head and tibial plateau were used to test the suitable plate and the appropriate screw length and orientation (Fig. 5). A surgeon that usually performed percutaneous surgery of tibial plateau and ankle fractures, sterilized the models and put them in the operating theater near the patient, to have an improved sense of spatial orientation for the percutaneous reduction of displaced fragments (Fig. 6).

All 3D printed models were used with 3D visualization tools to acquire the informed consent with the patients, showing and explaining his specific situation, the risk of specific fragment necrosis and to illustrate the surgical procedure. Patients reported an enthusiastic general appreciation about the use of this new technology in our hospitals. There was a substantial improvement in comprehension of the fracture before and after seeing the 3D printed models.

## Discussion and evaluation

Personalized medicine with 3d printing technology will be one of the most important fields of future medical research [11]. 3D printing is currently developing worldwide where physicians 3D print orthopedic disorders, tumors, and congenital pediatric problems. The use of 3d printed replica for facial surgery and neurosurgery is well known worldwide [7].

The application of 3D printing to plan articular-trauma surgery is not as common; this gap maybe is due to the difficulties in organizing all the steps of the workflow.

The CT images must be acquired with thin collimation and the images should be reconstructed with less than one millimeter thickness. Otherwise the final 3D model may not have adequate spatial resolution.

The conversion to .stl file must be performed immediately after CT scan and directly sent to a 3D Printer situated in hospital or in a service nearby. The conversion is done directly with the CT-workstation or commercial Softwares like OrisiX or Mimics [8], with a physician trained on it.

With this workflow, the model is available for surgeons and patients usually in 12 h.

Professional 3D Printers can print in different materials. For medical models, physicians print in ABS, PLA with different color (white, transparent, red…),or VisiJet (like gypsum) differentiating anatomical parts and pathologies.

In our experience, we suggest the use of VisiJet material (colored or not) to reproduce bone fractures because the models are more realistic then with other material.

Alternately, a bone fracture printed in white ABS or PLA is an acceptable model for surgeon and patients.

There are differences between Software in conversion “DICOM to stl:” numerous factors including segmentation technique and STL generation algorithms could be a source of potential error and loosening of details in the final model [4, 5].

In future a more standardized process (physician/radiologist technician training, software algorithm segmentation, quality of printers…) must be applied to allow a safe use of these models in clinical practice worldwide.

In this multi-centric experience we notice that the use is these models is well appreciated by surgeons and patients and we are currently discussing to introduce the use of the 3d printed replica as a mandatory step for the surgical informed consent. We do not suggest to print simple or diaphyseal fractures, were first there is no indication to investigate the fractures with a CT scan (according to general good orthopedic practice). Further studies and cost analysis must be performed around this topic to investigate the feasibility of the process.

## Conclusion

3D printing of articular fractures are innovative procedures and generate models to achieve a real preoperative tangible evaluation of the fractures and procedures and to improve patients compliance and care. This application is a small step in the future of the personalized medicine and in the quality improvement of the health system.

The authors did not received grants or outside funding in support of their research or preparation of this manuscript. No benefits in any form have been received or will be received from a commercial party related directly or indirectly to the subject of this article.
